# Emotional demands and all-cause and diagnosis-specific long-term sickness absence: a prospective cohort study in Sweden

**DOI:** 10.1093/eurpub/ckad072

**Published:** 2023-05-04

**Authors:** Elisabeth Framke, Kristina Alexanderson, Jeppe Karl Sørensen, Jacob Pedersen, Ida E H Madsen, Reiner Rugulies, Kristin Farrants

**Affiliations:** National Research Centre for the Working Environment, Copenhagen, Denmark; Department of Neurology, The Danish Multiple Sclerosis Registry, Copenhagen University Hospital, Glostrup, Denmark; Division of Insurance Medicine, Department of Clinical Neuroscience, Karolinska Institutet, Stockholm, Sweden; National Research Centre for the Working Environment, Copenhagen, Denmark; National Research Centre for the Working Environment, Copenhagen, Denmark; National Research Centre for the Working Environment, Copenhagen, Denmark; National Research Centre for the Working Environment, Copenhagen, Denmark; Section of Epidemiology, Department of Public Health, University of Copenhagen, Copenhagen, Denmark; Division of Insurance Medicine, Department of Clinical Neuroscience, Karolinska Institutet, Stockholm, Sweden

## Abstract

**Background:**

High emotional demands at work require sustained emotional effort and are associated with adverse health outcomes. We tested whether individuals in occupations with high emotional demands, compared with low demands, had a higher future risk of all-cause long-term sickness absence (LTSA). We further explored whether the risk of LTSA associated with high emotional demands differed by LTSA diagnoses.

**Methods:**

We conducted a prospective, nationwide cohort study on the association between emotional demands and LTSA (>30 days) in the workforce in Sweden (*n* = 3 905 685) during a 7-year follow-up. Using Cox regression, we analyzed sex-stratified risks of all-cause and diagnosis-specific LTSA due to common mental disorders (CMD), musculoskeletal disorders (MSD) and all other diagnoses. Multivariable adjusted models included age, birth country, education, living area, family situation and physical work demands.

**Results:**

Working in emotionally demanding occupations was associated with a higher risk of all-cause LTSA in women [hazard ratio (HR) = 1.92, 95% confidence interval (CI): 1.88–1.96] and men (HR = 1.23, 95% CI: 1.21–1.25). In women, the higher risk was similar for LTSA due to CMD, MSD and all other diagnoses (HR of 1.82, 1.92 and 1.93, respectively). In men, risk of LTSA due to CMD was pronounced (HR = 2.01, 95% CI: 1.92–2.11), whereas risk of LTSA due to MSD and all other diagnoses was only slightly elevated (HR of 1.13, both outcomes).

**Conclusions:**

Workers in occupations with high emotional demands had a higher risk of all-cause LTSA. In women, risk of all-cause and diagnosis-specific LTSA were similar. In men, the risk was more pronounced for LTSA due to CMD.

## Introduction

Dealing with sick or dying patients, taking care of clients’ emotional needs, responding to clients’ sorrows and worries or handling aggressive customers entails high emotional demands at work.^1^^,^^2^ Emotional demands at work require a sustained emotional effort^3^^,^^4^ and have been linked to a higher risk of long-term sickness absence (LTSA).^5–8^

Most previous studies on emotional demands at work and LTSA have been limited by using self-reported data on emotional demands and by analyzing smaller study populations.^6–9^ The use of self-reported exposure measurement may be problematic as the experience of emotional demands may be influenced by the affective state of the respondent.^2^^,^^10^ Small study samples may be vulnerable to selection bias and may have low statistical power. Studies of emotional demands and LTSA in the working populations of Denmark, Norway, Netherlands and Europe were conducted based on 3188; 6758; 31 884 and 32 708 respondents, respectively. All four studies had non-response rates about 40%.^6–9^ Furthermore, all but one^9^ of the previous studies on emotional demands at work and LTSA have not included the LTSA diagnoses and were restricted to examine all-cause LTSA. This is a limitation, as diagnosis-specific LTSA may help to understand through which pathways—e.g. psychological, psychobiological or behavioural—emotional demands may affect workers’ health. The only study that included LTSA diagnoses^9^ reported a higher risk of LTSA due to a mental diagnosis, after adjustment for other psychosocial work factors.

A nationwide, complete cohort study from Denmark reported a higher risk of LTSA among employees in jobs with a high level of emotional demands compared with employees in jobs with a low level of emotional demands.^11^ The study measured emotional demands at work with a job exposure matrix (JEM), i.e. exposure data were not on the individual level but aggregated on the job group level. The study was limited, though, by analyzing all-cause LTSA only, as diagnoses are not available in Danish sickness absence registers. In Sweden, sickness absence registers include diagnoses, however, a Swedish JEM on emotional demands has not yet been developed, to our knowledge. Therefore, in the present study, we used the Danish JEM on emotional demands at work^11^ and applied this JEM to Swedish register data on working populations and their sickness absence. This allowed us analysing not only all-cause LTSA but also diagnosis-specific LTSA, in terms of LTSA due to common mental disorders (CMD) and musculoskeletal disorders (MSD), respectively. CMD and MSD are the two leading categories of LTSA diagnoses in high-income countries,^12^ including Sweden.^13^

The aim of this study was to test the hypothesis that women and men in occupations with high emotional demands at work, compared with low demands, had a higher risk of future all-cause LTSA. In addition, we explored whether this differed by LTSA diagnoses in terms of CMD, MSD and all other diagnoses except CMD and MSD, respectively.

## Methods

We conducted a nationwide 7-year prospective cohort study on the association between emotional demands at work and the future risk of LTSA in the workforce in Sweden. We examined the risk of all-cause LTSA and risk of LTSA due to CMD, due to MSD and due to all diagnoses except CMD and MSD.

### Data

We used pseudoanonymized individual-level data from the Longitudinal Integration Database for Health Insurance and Labour Market Studies (LISA by Swedish acronym) held by Statistics Sweden for information on the study population, occupational group, covariates, old-age pension and emigration. Information on LTSA (diagnoses and dates) and disability pension was obtained from Microdata for the Analysis of Social Insurance (MIDAS by Swedish acronym) held by the Swedish Social Insurance Agency. Information on date of death was retrieved from the Cause of Death Register kept by the National Board of Health and Welfare. For information on emotional demands at work, we used a Danish psychosocial JEM.^11^

### Study population

In total, 5 145 621 individuals aged 18–60 years were registered as living in Sweden both in December 2008 and December 2009 (the entire year of 2009). The study population (3 905 685 individuals; 1 841 250 women and 2 064 435 men), consisted of those who had no disability pension and no LTSA defined as more than 30 days in the year 2009 (baseline), had an income from work high enough to qualify for sickness absence benefits [≥10 176 SEK (=987 Euros by the 2009 conversion rate)] and had an occupational classification code.

### Exposure to emotional demands at work

We measured emotional demands at work in 2009 with a JEM based on information from the Danish Work Environment Cohort Study (DWECS). The construction of the Danish emotional demands at work JEM is described in detail elsewhere.^11^ Briefly, DWECS is a survey about working conditions and health in Denmark, conducted in a nationwide sample of the Danish workforce from 1990 to 2010.^14^^,^^15^ DWECS includes three items on emotional demands at work, each rated on a five-point scale: (i) Does your work put you in emotionally disturbing situations? (ii) Is your work emotionally demanding? (iii) Do you get emotionally involved in your work?

Using data from the 2000 and 2005 waves of DWECS, we estimated the JEM as the predicted level of emotional demands at work given job group [coded according to DISCO-88,^16^ the Danish version of the International Standard Classification of Occupations (ISCO)-88 system^17^] sex, age and year of data collection (2000, 2005). Next, we translated Swedish occupational codes, Swedish Standard for Occupational Classification (SSYK-96),^18^ the Swedish version of ISCO-88 codes, into the corresponding DISCO-88 codes. We then assigned the predicted levels of emotional demands at work to each individual in the study population at baseline in 2009, according to their occupational group, sex and age. Finally, we categorized individuals into groups with low, medium-low, medium-high and high levels of emotional demands at work based on a quartile split of the distribution in the study population.

### The public sickness absence system in Sweden

All individuals living in Sweden, aged 16 years and above, are covered by public sickness absence insurance in case of reduced work capacity due to disease or injury, provided that the individual has an income from work, parental leave benefits or unemployment benefits. The first day of a sickness absence spell is a waiting day and the following 13 days are covered by the employer. Sickness absence days from day 15 onwards are covered by the Social Insurance Agency, which also covers benefits from day 2 onwards for unemployed individuals.^13^ After the 7th day, a medical certificate is required, issued by the physician responsible for assessing the patient’s work capacity. The certificate includes the diagnosis leading to the work incapacity, coded according to the International Classification of Diseases, 10th revision (ICD-10).

### LTSA diagnoses

We defined LTSA as having had a sickness absence spell of >30 days between 1 January 2010 and 31 December 2016. First, we coded all-cause LTSA as LTSA due to all sickness absence diagnoses. Next, using the ICD-10 codes on the sickness absence certificates, we constructed three categories of LTSA: (i) CMD [depressive disorders (F32, F33), anxiety disorders (F40, F41), reaction to severe stress and adjustment disorders (F43) and problems related to life management difficulty, including burnout (Z73)]; (ii) MSD (M00-M99) and (iii) all other diagnoses including those with missing information on diagnosis.

### Covariates

All covariates were measured at baseline in December 2009. As covariates, we included age (categorized as 18–24, 25–34, 35–44, 45–54, 55–60 years); country of birth [Sweden, other Nordic country, other EU27, rest of world (including missing)]; type of living area [large city (Stockholm, Gothenburg, Malmö with surrounding municipalities), medium-sized town (municipalities with >90 000 inhabitants within 30 km of municipal centre), small town or rural (municipalities with <90 000 inhabitants within 30 km of municipal centre)]; family situation (married/cohabiting with children <18 years living at home, married/cohabiting with no children <18 years living at home, single with children <18 years living at home, single with no children <18 years living at home); educational level [elementary or equivalent (≤9 years or missing), high school or equivalent (10–12 years), university/college or equivalent (>12 years)] and, finally, physical demands at work (measured with a JEM, obtained in a similar way from DWECS as we obtained the JEM for emotional demands^11^ and categorized into quartiles based on the distribution in the study population).

### Statistical analysis

All analyses were sex-stratified using SAS 9.4. We analyzed the association between emotional demands at work at baseline and the risk of LTSA during the 7-year follow-up by estimating hazard ratios (HR) and 95% confidence intervals (95% CI) using Cox proportional hazards regression models with calendar time as the underlying time axis.

We followed each individual from 1 January 2010 until the first episode of LTSA or censoring due to disability pension, early old-age pension, emigration, death or end of follow-up (31 December 2016), whichever came first. First, we analyzed the association between emotional work demands and all-cause LTSA. Next, we analyzed the association between emotional demands and diagnosis-specific LTSA according to the three diagnostic groups: CMD, MSD and all other diagnoses. In addition to the reasons for censoring mentioned above, for each of the three diagnosis-specific analyses, we also censored due to LTSA due to all other diagnoses than the diagnostic group analyzed, whichever came first.

We fitted three models. Estimates were unadjusted in model 1, adjusted for age in model 2, and further adjusted for birth country, type of living area, family situation, educational level and physical demands at work in model 3.

In sensitivity analyses, we repeated the main analyses while changing the definition of LTSA from a sickness absence spell of more than 30 days to a sickness absence spell of more than 14 days. In post-hoc analyses, we explored the reason for the changes in the estimates from model 2 to model 3. This project was approved by the Regional Ethical Review Board in Stockholm.

### Role of the funding source

The funders of the study had no role in study design, data collection, data analysis, data interpretation, writing of the report or decision to submit for publication. E.F., J.P., K.A. and K.F. had full access to all data. E.F., K.A., R.R. and K.F. had final responsibility for the decision to submit for publication.

## Results

### Characteristics of the study population

Table 1 shows the baseline characteristics of the study population in the year 2009, separately for the 1 841 250 women and 2 064 435 men. About 86% in both sexes were born in Sweden. Most had at least some high school education and were either single without children or married or cohabitant with children. In total, about three quarters of the population were living in big or medium-sized cities.

**Table 1:** Characteristics of the study population at baseline in 2009, separately for women and men

		Women	Men	
		*n* (%)	*n* (%)	
Sex	Women	1 841 250 (47.1)		
	Men			2 064 435 (52.9)
Age	18–24 years	283 189 (15.4)		286 963 (13.9)
	25–34 years	412 993 (22.4)		474 756 (23.0)
	35–44 years	488 449 (26.5)		556 170 (26.9)
	45–54 years	431 374 (23.4)		488 278 (23.7)
	55–60 years	225 245 (12.2)		258 268 (12.5)
Birth country	Sweden	1 585 467 (86.1)		1 782 669 (86.4)
	Other Nordic countries	46 424 (2.5)		39 632 (1.9)
	Other EU27 countries	46 061 (2.5)		50 861 (2.5)
	The rest of the world	163 298 (8.9)		191 273 (9.3)
Education	Elementary	173 278 (9.4)		296 340 (14.4)
	High school	862 239 (46.8)		1 074 714 (52.1)
	University/college	805 733 (43.8)		693 381 (33.6)
Living area	Big cities	724 270 (39.3)		788 902 (38.2)
	Medium-sized cities	648 179 (35.2)		728 914 (35.3)
	Small town/rural areas	468 801 (25.5)		546 619 (26.5)
Family situation	Married or cohabitant without children	331 643 (18.0)		307 502 (14.9)
	Married or cohabitant with children	647 039 (35.1)		725 680 (35.2)
	Single without children	722 444 (39.2)		988 168 (47.9)
	Single with children	140 124 (7.6)		43 085 (2.1)
Physical demands	First quartile	482 208 (26.2)		663 987 (32.2)
	Second quartile	513 616 (27.9)		379 408 (18.4)
	Third quartile	476 970 (25.9)		465 055 (22.5)
	Fourth quartile	368 456 (20.0)		555 985 (26.9)

### Emotional demands and all-cause LTSA

Among women, during 9 904 271 person-years of follow-up, we identified 269 280 cases of incident all-cause LTSA (27.2 per 1000 person-years). Among men, during 12 300 943 person-years of follow-up, we identified 237 184 cases of incident all-cause LTSA (19.3 per 1000 person-years).

Table 2 shows person-years, LTSA cases, LTSA cases per 1000 person-years, the unadjusted (model 1), the minimal (age-) adjusted (model 2) and the most-adjusted (model 3) estimates for the associations between emotional demands and all-cause and diagnosis-specific LTSA in women and men. Notable is that the number of person-years in employees in occupations with high emotional demands is three times higher among women than among men.

**Table 2:** Emotional demands at work and risk of a new spell of long-term sickness absence exceeding 30 days during 7 years of follow-up

			PY	Cases	Cases per 1000 PY	Model 1	Model 2	Model 3
						HR (95% CI)	HR (95% CI)	HR (95% CI)
LTSA due to all causes								
	Women							
		Low	1 503 561	30 063	20.0	1.00	1.00	1.00
		Medium-low	2 706 005	61 572	22.8	1.12 (1.11–1.14)	1.09 (1.07–1.11)	1.23 (1.21–1.25)
		Medium-high	3 771 867	69 452	18.4	0.92 (0.90–0.93)	0.90 (0.88–0.91)	1.32 (1.30–1.34)
		High	3 930 120	108 193	27.5	1.34 (1.32–1.35)	1.30 (1.27–1.32)	1.92 (1.88–1.96)
	Men							
		Low	5 896 703	114 974	19.5	1.00	1.00	1.00
		Medium-low	3 976 768	66 309	16.7	0.85 (0.84–0.85)	0.71 (0.70–0.72)	0.91 (0.90–0.92)
		Medium-high	3 500 485	37 473	10.7	0.56 (0.55–0.56)	0.46 (0.45–0.46)	0.82 (0.81–0.83)
		High	1 169 682	18 428	15.8	0.80 (0.78–0.81)	0.64 (0.63–0.66)	1.23 (1.21–1.25)
LTSA due to CMD								
	Women							
		Low	1 503 561	6529	4.3	1.00	1.00	1.00
		Medium-low	2 706 005	11 942	4.4	1.01 (0.98–1.04)	1.15 (1.11–1.19)	1.20 (1.16–1.25)
		Medium-high	3 771 867	15 139	4.0	0.91 (0.88–0.94)	1.06 (1.03–1.11)	1.31 (1.26–1.37)
		High	3 930 120	21 219	5.4	1.20 (1.17–1.24)	1.46 (1.41–1.52)	1.82 (1.75–1.90)
	Men							
		Low	5 896 703	12 950	2.2	1.00	1.00	1.00
		Medium-low	3 976 768	7900	2.0	0.91 (0.88–0.93)	0.93 (0.90–0.96)	1.04 (1.01–1.08)
		Medium-high	3 500 485	6201	1.8	0.83 (0.80–0.86)	0.86 (0.83–0.89)	1.17 (1.13–1.22)
		High	1 169 682	3632	3.1	1.38 (1.33–1.44)	1.44 (1.38–1.50)	2.01 (1.92–2.11)
LTSA due to MSD								
	Women							
		Low	1 503 561	5187	3.4	1.00	1.00	1.00
		Medium-low	2 706 005	13 314	4.9	1.38 (1.34–1.43)	0.94 (0.90–0.98)	1.14 (1.09–1.19)
		Medium-high	3 771 867	13 391	3.6	1.03 (1.00–1.07)	0.66 (0.64–0.69)	1.21 (1.15–1.26)
		High	3 930 120	24 335	6.2	1.73 (1.68–1.79)	1.04 (1.00–1.08)	1.92 (1.83–2.01)
	Men							
		Low	5 896 703	30 736	5.2	1.00	1.00	1.00
		Medium-low	3 976 768	18 547	4.7	0.89 (0.87–0.91)	0.67 (0.65–0.68)	0.88 (0.86–0.90)
		Medium-high	3 500 485	9177	2.6	0.52 (0.51–0.53)	0.38 (0.37–0.39)	0.77 (0.74–0.79)
		High	1 169 682	4472	3.8	0.73 (0.70–0.75)	0.52 (0.50–0.54)	1.13 (1.08–1.17)
LTSA due to all causes except CMD and MSD								
	Women							
		Low	1 503 561	18 347	12.2	1.00	1.00	1.00
		Medium-low	2 706 005	36 316	13.4	1.09 (1.07–1.11)	1.10 (1.08–1.13)	1.25 (1.22–1.28)
		Medium-high	3 771 867	40 922	10.8	0.89 (0.87–0.90)	0.92 (0.90–0.94)	1.34 (1.31–1.38)
		High	3 930 120	62 639	15.9	1.27 (1.25–1.30)	1.32 (1.29–1.34)	1.93 (1.88–1.98)
	Men							
		Low	5 896 703	71 288	12.1	1.00	1.00	1.00
		Medium-low	3 976 768	39 862	10.0	0.82 (0.81–0.83)	0.69 (0.68–0.70)	0.89 (0.88–0.91)
		Medium-high	3 500 485	22 095	6.3	0.52 (0.52–0.53)	0.43 (0.43–0.44)	0.79 (0.77–0.80)
		High	1 169 682	10 324	8.8	0.72 (0.70–0.74)	0.59 (0.57–0.60)	1.13 (1.11–1.16)

*Notes*: Model 1: Unadjusted. Model 2: Adjusted for age. Model 3: Further adjusted for birth country, education, type of living area, family situation and physical demands at work.

Abbreviations: LTSA, long-term-sickness absence; CMD, common mental disorder; MSD, musculoskeletal disorder; PY, person-years; HR, hazard ratio; CI, confidence interval.

Among women, high emotional demands were associated with a higher risk of all-cause LTSA in all three models, with HRs of 1.34 (unadjusted, model 1), 1.30 (age-adjusted, model 2) and 1.92 (most-adjusted, model 3), respectively. Among men, high emotional demands were associated with a lower risk of LTSA in model 1 (HR = 0.80) and model 2 (HR = 0.64) and with a higher risk of LTSA in model 3 (HR = 1.23).

**Figure 1:**
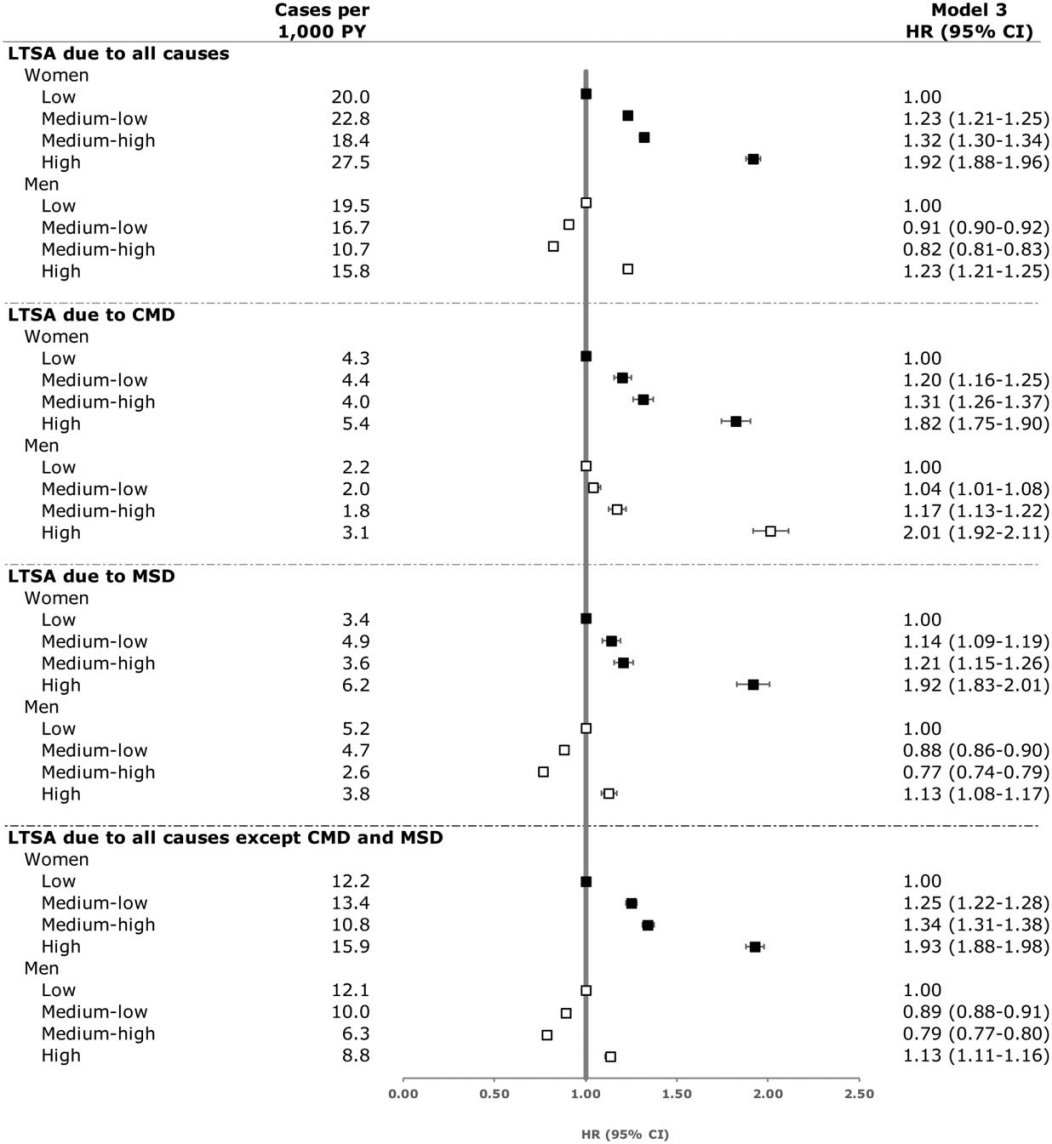
Emotional demands at work and risk of a new spell of long-term sickness absence exceeding 30 days during 7 years of follow-up. Model 3: Adjusted for age, birth country, education, type of living area, family situation and physical demands at work. Abbreviations: LTSA, long-term-sickness absence; CMD, common mental disorder; MSD, musculoskeletal disorder; PY, person-years; HR, hazard ratio; CI, confidence interval.

Figure 1 depicts the most-adjusted estimates (HR and 95% CI for model 3) for both all-cause and diagnosis-specific LTSA.

### Emotional demands and diagnosis-specific LTSA

Of the 269 280 incident episodes of all-cause LTSA during follow-up in women, 54 829 (20%) were due to CMD (5.5 per 1000 person-years), 56 227 (21%) were due to MSD (5.7 per 1000 person-years), while 158 224 (59%) were due to other diagnoses (16.0 per 1000 person-years). Of the 237 184 incident episodes of all-cause LTSA during follow-up in men, 30 683 (13%) were due to CMD (2.5 per 1000 person-years), 62 932 (27%) were due to MSD (5.1 per 1000 person-years) and 143 569 (61%) were due to other diagnoses (11.7 per 1000 person-years).

#### Common mental disorders

In the most-adjusted-model (model 3), compared with working in occupations with low emotional demands, working in occupations with medium-low (HR = 1.20, 95% CI: 1.16–1.25), medium-high (HR = 1.31, 95% CI: 1.26–1.37) and high emotional demands (HR = 1.82, 95% CI: 1.75–1.90) was associated with a higher risk of LTSA due to CMD (table 2 and figure 1). Among men, compared with working in occupations with low emotional demands, working in occupations with medium-low (HR = 1.04, 95% CI: 1.01–1.08), medium-high (HR = 1.17, 95% CI: 1.13–1.22) and high emotional demands (HR = 2.01, 95% CI: 1.92–2.11) was also associated with a higher risk of LTSA due to CMD (table 2 and figure 1).

In the age-adjusted model (model 2), emotional demands were also associated with a higher risk of LTSA due to CMD among both women and men; however, associations were weaker than in the most-adjusted model (table 2).

#### Musculoskeletal diagnoses

In the most-adjusted model, among women, compared with working in occupations with low emotional demands, working in occupations with medium-low (HR = 1.14, 95% CI: 1.09–1.19), medium-high (HR = 1.21, 95% CI: 1.15–1.26) and high emotional demands (HR = 1.92, 95% CI: 1.83–2.01) was associated with a higher risk of LTSA due to MSD. Among men, compared with working in occupations with low emotional demands, working in occupations with medium-low (HR = 0.88, 95% CI: 0.86–0.90) and medium-high emotional demands (HR = 0.77, 95% CI: 0.74–0.79) was associated with a lower risk of LTSA due to MSD, whereas working in occupations with high emotional demands was associated with a higher risk of LTSA due to MSD (HR = 1.13, 95% CI: 1.08–1.17).

In the age-adjusted model (model 2), among women, emotional demands were associated with a higher risk of LTSA due to MSD (albeit with a weaker estimate than in the most-adjusted model), whereas among men, high emotional demands were associated with a lower risk of LTSA (table 2).

#### All other diagnoses except CMD and MSD

In the most-adjusted model, among women, compared with working in occupations with low emotional demands, working in occupations with medium-low (adjusted HR = 1.25, 95% CI: 1.22–1.28), medium-high (adjusted HR = 1.34, 95% CI: 1.31–1.38) and high emotional demands (adjusted HR = 1.93, 95% CI: 1.88–1.98) was associated with a higher risk of LTSA due to all other diagnoses, except CMD and MSD. Among men, compared with working in occupations with low emotional demands, working in occupations with medium-low (adjusted HR = 0.89, 95% CI: 0.88–0.91) and medium-high emotional demands (adjusted HR = 0.79, 95% CI: 0.77–0.80) was associated with a lower risk of LTSA due to all other diagnoses, whereas working in occupations with high emotional demands was associated with a higher risk of LTSA due to all diagnoses (adjusted HR = 1.13, 95% CI: 1.11–1.16).

In the age-adjusted model (model 2), among women, emotional demands were associated with a higher risk of LTSA due to all other causes (albeit with a weaker estimate than in the most-adjusted model), whereas among men, high emotional demands were associated with a lower risk of LTSA (table 2).

### Sensitivity analyses

When repeating the main analyses while defining LTSA as sickness absence spells with a duration of more than 14 days instead of sickness absence spells of more than 30 days, results were similar (results shown in Supplementary table S1 and Supplementary figure S1).

### Post-hoc analyses

*Post-hoc* analyses revealed that the main reason for the changes in the estimates from model 2 to model 3 was the adjustment for physical demands at work (Supplementary table S2). After adding physical demands as a covariate, the association between high emotional demands and risk of LTSA became stronger in all analyses. In men, in some analyses, the estimate for emotional demands even changed direction, from being associated with a lower risk of LTSA before adjustment to a higher risk of LTSA after adjustment.

## Discussion

In this prospective, nationwide and complete cohort study in Sweden, we found that working in emotionally demanding jobs was associated with a higher risk of future all-cause LTSA in both women and men. The risk of LTSA did not differ by diagnosis in women. In men, exposure to emotional demands was associated with a high risk of LTSA due to CMD, whereas risk of LTSA due to MSD and due to all other diagnoses was only slightly elevated.

The findings on all-cause LTSA are in agreement with previous studies,^5–7^^,^^11^ reporting that emotional demands at work are a risk factor for all-cause LTSA. To our knowledge, previously only one study has examined the risk of diagnosis-specific LTSA in relation to exposure to emotional demands at work.^9^ Van Hoffen et al.^9^ linked survey data of 31 884 non-sick-listed employees in the Netherlands to diagnosis-specific register-based sickness absence data. Adverse psychosocial working conditions were associated with a higher risk of LTSA due to mental diagnoses, and after adjusting for other psychosocial work factors the association between emotional demands and LTSA due to mental diagnoses was the strongest. In this study, we did not account for other psychosocial factors at work. Other JEM-based studies of emotional demands and depressive disorder^19^ and all-cause LTSA^11^ have accounted for other work-related psychosocial factors and found that estimates for emotional demands were robust for this adjustment.

In our study, women had more than three times more person-years in occupations with high emotional demands than men. Some of the associations between emotional demands and LTSA differed between women and men. In women, the risk was higher in all diagnostic groups with risk estimates of approximately equal magnitude, whereas in men, the risk was higher for LTSA due to CMD and only slightly elevated for the other diagnostic groups. We do not know the reasons for these different patterns between women and men across LTSA diagnoses. One explanation could be that the psychological, biological and behavioural mechanism through which emotional demands affect workers’ health may be partly different for women and men. This should be examined in further studies.

*Post-hoc* analyses showed that after adjustment for high physical demands at work, the estimates for the association between high emotional demands and risk of LTSA became stronger and in some analyses (among men) even changed direction from a lower to a higher risk of LTSA. Thus, high physical demands seem to confound (mask) the association between high emotional demands and risk of LTSA. We recommend to routinely adjust for high physical demands in future studies on emotional demands results highlight the need that employers, public health decision makers and physicians understand the role of emotional demands at work as a potential contributor to sickness absence and that organizational policies are developed to help workplaces to handle this stressor.^20^ Such policies in relation to emotional demands have, however, not yet been tested in intervention studies. It may be debatable whether reduction of exposure to emotional demands at work is possible, as emotional demands may be considered part of the job in several occupations. Identifying other modifiable work factors that may modify possible harmful effects of work-related emotional demands may therefore be important. Previous research suggests that increasing possibilities for development and reducing work-related role conflicts,^11^ but not increasing leadership quality or influence or reducing physical demands,^5^^,^^11^ may modify harmful effects of emotional demands at work. Intervention studies, including randomized controlled trials, are needed on these and other factors to evaluate the effectiveness of interventions.

### Strengths and limitations

The strengths of this study include: First, the nationwide and complete cohort of all 3.9 million women and men aged 18–60 years in paid work in Sweden without LTSA or disability pension at baseline, thereby circumventing possible selection bias. Second, the use of non-self-reported measurement of emotional demands at work and of administrative data on covariates and LTSA not affected by recall or reporting bias. Third, there were no drop-outs during the 7-year follow-up. Fourth, the availability of information on LTSA-diagnoses, provided by the patient’s treating physician.

The study also has some limitations. First, JEM’s may led to exposure misclassification as they disregard heterogeneity in exposure between workers with the same job code. Therefore, interpretations should be made on the level of occupations (i.e. risk of LTSA in job groups with specific average exposure levels of emotional demands) and caution is needed when interpreting results on the individual level.^21^ Second, this is an observational study and therefore bias by residual confounding, including by other psychosocial work factors, cannot be ruled out. However, previous studies have shown that emotional demands remained as a risk factor for all-cause LTSA, LTSA due to mental diagnoses, and depressive disorder, also after adjusting for other psychosocial factors.^9^^,^^11^^,^^19^ Third, we applied a JEM developed based on Danish data in a cohort of the workforce in Sweden, as a Swedish JEM on emotional demands was not available. Fourth, generalizability may be a limitation. Assuming that the Danish JEM for emotional demands at work can reasonably be applied in a Swedish context, we consider our results generalizable to the workforce in Sweden. Since administrative systems of LTSA differ from one country to another, however, our results may not be generalizable to countries with other types of welfare systems.

### Conclusion

Workers in occupations with high emotional demands had a higher risk of all-cause LTSA. In women, risk of all-cause and diagnosis-specific LTSA were similar. In men, the risk was more pronounced for LTSA due to CMD. *Post-hoc* analyses suggested that the association between emotional demands and risk of LTSA were partially masked when analyses were not adjusted for physical demands at work.

## Supplementary Material

ckad072_Supplementary_Data

## Data Availability

The data cannot be made publicly available, according to privacy regulations. According to the General Data Protection Regulation, the Swedish law SFS 2018:218, the Swedish Data Protection Act, the Swedish Ethical Review Act and the Public Access to Information and Secrecy Act, data can only be made available, after legal review, for researchers who meet the criteria for access to this type of sensitive and confidential data. Readers may contact Professor Kristina Alexanderson (kristina.alexanderson@ki.se) regarding the data. Workers who are doing person-related work, e.g. dealing with sick or dying patients, taking care of clients’ emotional needs, responding to clients’ sorrows and worries, and handling aggressive customers are exposed to emotional demands at work. Workers in occupations with a high level of emotional demands at work had a higher risk of all-cause and diagnosis-specific long-term sickness absence. Among women, high emotional demands at work were associated with a markedly higher risk of long-term sickness absence for all examined diagnostic categories, whereas among men, the risk was particularly pronounced for long-term sickness absence due to common mental disorders. Dealing with high emotional demands at work may be key in preventing long-term sickness absence, especially long-term sickness absence due to common mental disorders.

## References

[1] Zapf D , Seifert C, Schmutte B, et al Emotion work and job stressors and their effects on burnout. Psychol Health 2001;16:527–45. 22804497 10.1080/08870440108405525

[2] Vammen MA , Mikkelsen S, Forman JL, et al Emotional demands and exhaustion: cross-sectional and longitudinal associations in a cohort of Danish public sector employees. Int Arch Occup Environ Health 2019;92:639–50. 30864025 10.1007/s00420-018-01398-w

[3] Zapf D. Emotion work and psychological well-being: a review of the literature and some conceptual considerations. Hum Resour Manag Rev 2002;12:237–68.

[4] Vegchel NV , Jonge JD, Söderfeldt M, et al Quantitative versus emotional demands among Swedish human service employees: moderating effects of job control and social support. Int J Stress Manag 2004;11:21–40.

[5] Rugulies R , Sørensen JK, Madsen IEH, et al Can leadership quality buffer the association between emotionally demanding work and risk of long-term sickness absence? Eur J Public Health 2021;31:739–41. 34219170 10.1093/eurpub/ckab090 PMC8561255

[6] Aagestad C , Johannessen HA, Tynes T, et al Work-related psychosocial risk factors for long-term sick leave: a prospective study of the general working population in Norway. J Occup Environ Med 2014;56:787–93. 25099403 10.1097/JOM.0000000000000212

[7] Slany C , Schütte S, Chastang JF, et al Psychosocial work factors and long sickness absence in Europe. Int J Occup Environ Health 2014;20:16–25. 24176393 10.1179/2049396713Y.0000000048 PMC4137803

[8] Rugulies R , Aust B, Pejtersen JH. Do psychosocial work environment factors measured with scales from the Copenhagen Psychosocial Questionnaire predict register-based sickness absence of 3 weeks or more in Denmark? Scand J Public Health 2010;38:42–50. 21172770 10.1177/1403494809346873

[9] van Hoffen MFA , Rijnhart JJM, Norder G, et al Distress, work satisfaction, and work ability are mediators of the relation between psychosocial working conditions and mental health-related long-term sickness absence. J Occup Rehabil 2021;31:419–30. 33074455 10.1007/s10926-020-09931-w PMC8172497

[10] Framke E , Sørensen JK, Nordentoft M, et al Perceived and content-related emotional demands at work and risk of long-term sickness absence in the Danish workforce: a cohort study of 26 410 Danish employees. Occup Environ Med 2019;76:895–900. 31662424 10.1136/oemed-2019-106015 PMC6902065

[11] Framke E , Sørensen JK, Alexanderson K, et al Emotional demands at work and risk of long-term sickness absence in 1·5 million employees in Denmark: a prospective cohort study on effect modifiers. Lancet Public Health 2021;6:e752–9. 34563282 10.1016/S2468-2667(21)00185-7

[12] Janssens H , Clays E, De Clercq B, et al The relation between psychosocial risk factors and cause-specific long-term sickness absence. Eur J Public Health 2014;24:428–33. 24567292 10.1093/eurpub/cku009

[13] Sweden Social Insurance Agency. Social insurance in figures 2019 [Internet]. Available at: www.forsakringskassan.se/statistik (26 April 2023, date last accessed).

[14] Burr H , Bjorner JB, Kristensen TS, et al Trends in the Danish work environment in 1990-2000 and their associations with labor-force changes. Scand J Work Environ Health 2003;29:270–9. 12934720 10.5271/sjweh.731

[15] Feveile H , Olsen O, Burr H, Bach E. Danish Work Environment Cohort Study 2005: from idea to sampling design. Stat Transit 2007;8:441–58.

[16] Publikation: DISCO-88 1988 – Danmarks Statistik [Internet]. Available at: https://www.dst.dk/da/Statistik/nyheder-analyser-publ/Publikationer/VisPub?cid=4831 (26 April 2023, date last accessed).

[17] Bureau of Statistics, work unit of the Policy Integration Department [Internet]. Available at: https://www.ilo.org/public/english/bureau/stat/isco/isco88/index.htm (26 April 2023, date last accessed).

[18] Statistics Sweden. Meddelanden i samordningsfrågor för Sveriges officiella statistik [Internet]. Available at: https://share.scb.se/ov9993/data/publikationer/statistik/_publikationer/ov9999_1998a01_br_x70öp9803.pdf (26 April 2023, date last accessed).

[19] Madsen IEH , Sørensen JK, Bruun JE, et al Emotional demands at work and risk of hospital-treated depressive disorder in up to 1.6 million Danish employees: a prospective nationwide register-based cohort study. Scand J Work Environ Health 2022;48:302–11. 35262742 10.5271/sjweh.4020 PMC9524161

[20] Duchaine CS , Aubé K, Gilbert-Ouimet M, et al Psychosocial stressors at work and the risk of sickness absence due to a diagnosed mental disorder: a systematic review and meta-analysis. JAMA Psychiatry 2020;77:842–51. 32236498 10.1001/jamapsychiatry.2020.0322 PMC7113841

[21] Peters S. Although a valuable method in occupational epidemiology, job-exposure-matrices are no magic fix. Scand J Work Environ Health 2020;46:231–4. 32356897 10.5271/sjweh.3894

