# Supplementary material for: Emotional demands and all-cause and diagnosis-specific long-term sickness absence: a prospective cohort study in Sweden
Source: Eur J Public Health. 2023 May 4;33(3):435–41. doi: 10.1093/eurpub/ckad072 (PMC10234643; doi:10.1093/eurpub/ckad072)
Supplement: ckad072_Supplementary_Data [file ckad072_supplementary_data.docx]

**e-Appendix: Online Data Supplements for the article “Emotional demands and all-cause and diagnosis-specific long-term sickness absence: a prospective cohort study in Sweden”**

Elisabeth Framke, PhD (1, 2); Kristina Alexanderson, PhD (3); Jeppe Karl Sørensen, MSc (1); Jacob Pedersen, PhD (1); Ida EH Madsen, PhD (1); Reiner Rugulies, PhD (1, 4); Kristin Farrants, PhD (3)

1. National Research Centre for the Working Environment, Copenhagen, Denmark
2. The Danish Multiple Sclerosis Registry, Copenhagen University Hospital, Copenhagen, Denmark
3. Division of Insurance Medicine, Department of Clinical Neuroscience, Karolinska Institutet, SE-171 77 Stockholm, Sweden
4. Section of Epidemiology, Department of Public Health, University of Copenhagen, Copenhagen, Denmark

**e-Table 1: Emotional demands at work and risk of a new spell of long-term sickness absence exceeding 14 days during 7 years of follow-up**

|  |  |  | **PY** | **Cases** | **Cases per  1,000 PY** | **Model 1**  **HR (95% CI)** |  | **Model 2**  **HR (95% CI)** |  | **Model 3**  **HR (95% CI)** |
| --- | --- | --- | --- | --- | --- | --- | --- | --- | --- | --- |
| **LTSA due to all causes** | | |  |  |  |  |  |  |  |  |
|  | Women | |  |  |  |  |  |  |  |  |
|  |  | Low | 1,429,163 | 47,048 | 32.9 | 1.00 |  | 1.00 |  | 1.00 |
|  |  | Medium-low | 2,515,961 | 101,650 | 40.4 | 1.20 (1.18-1.21) |  | 1.08 (1.06-1.09) |  | 1.21 (1.20-1.23) |
|  |  | Medium-high | 3,524,193 | 120,748 | 34.3 | 1.02 (1.01-1.03) |  | 0.91 (0.90-0.92) |  | 1.30 (1.28-1.31) |
|  |  | High | 3,561,626 | 187,424 | 52.6 | 1.53 (1.52-1.55) |  | 1.34 (1.33-1.36) |  | 1.91 (1.88-1.93) |
|  | Men | |  |  |  |  |  |  |  |  |
|  |  | Low | 5,592,694 | 180,279 | 32.2 | 1.00 |  | 1.00 |  | 1.00 |
|  |  | Medium-low | 3,786,582 | 106,303 | 28.1 | 0.87 (0.86-0.87) |  | 0.72 (0.71-0.72) |  | 0.91 (0.90-0.92) |
|  |  | Medium-high | 3,387,136 | 61,366 | 18.1 | 0.57 (0.56-0.57) |  | 0.46 (0.46-0.47) |  | 0.79 (0.78-0.80) |
|  |  | High | 1,115,254 | 30,222 | 27.1 | 0.84 (0.83-0.85) |  | 0.67 (0.66-0.68) |  | 1.21 (1.20-1.23) |
| **LTSA due to CMD** | | |  |  |  |  |  |  |  |  |
|  | Women | |  |  |  |  |  |  |  |  |
|  |  | Low | 1,429,163 | 8,796 | 6.2 | 1.00 |  | 1.00 |  | 1.00 |
|  |  | Medium-low | 2,515,961 | 16,633 | 6.6 | 1.05 (1.03-1.08) |  | 1.14 (1.10-1.17) |  | 1.19 (1.15-1.23) |
|  |  | Medium-high | 3,524,193 | 21,779 | 6.2 | 0.99 (0.96-1.01) |  | 1.08 (1.05-1.11) |  | 1.31 (1.27-1.35) |
|  |  | High | 3,561,626 | 31,617 | 8.9 | 1.39 (1.36-1.43) |  | 1.57 (1.52-1.62) |  | 1.90 (1.84-1.97) |
|  | Men | |  |  |  |  |  |  |  |  |
|  |  | Low | 5,592,694 | 17,394 | 3.1 | 1.00 |  | 1.00 |  | 1.00 |
|  |  | Medium-low | 3,786,582 | 10,709 | 2.8 | 0.91 (0.89-0.93) |  | 0.92 (0.90-0.95) |  | 1.04 (1.01-1.07) |
|  |  | Medium-high | 3,387,136 | 8,646 | 2.6 | 0.83 (0.81-0.85) |  | 0.85 (0.82-0.87) |  | 1.12 (1.08-1.16) |
|  |  | High | 1,115,254 | 5,027 | 4.5 | 1.43 (1.39-1.48) |  | 1.47 (1.43-1.53) |  | 1.99 (1.91-2.07) |
| **LTSA due to MSD** | | |  |  |  |  |  |  |  |  |
|  | Women | |  |  |  |  |  |  |  |  |
|  |  | Low | 1,429,163 | 8,015 | 5.6 | 1.00 |  | 1.00 |  | 1.00 |
|  |  | Medium-low | 2,515,961 | 20,872 | 8.3 | 1.44 (1.40-1.48) |  | 0.93 (0.90-0.96) |  | 1.16 (1.12-1.19) |
|  |  | Medium-high | 3,524,193 | 21,212 | 6.0 | 1.06 (1.03-1.09) |  | 0.64 (0.62-0.66) |  | 1.21 (1.17-1.25) |
|  |  | High | 3,561,626 | 38,611 | 10.8 | 1.85 (1.81-1.90) |  | 1.05 (1.01-1.08) |  | 2.01 (1.94-2.08) |
|  | Men | |  |  |  |  |  |  |  |  |
|  |  | Low | 5,592,694 | 46,677 | 8.3 | 1.00 |  | 1.00 |  | 1.00 |
|  |  | Medium-low | 3,786,582 | 28,111 | 7.4 | 0.88 (0.87-0.90) |  | 0.66 (0.65-0.67) |  | 0.88 (0.87-0.90) |
|  |  | Medium-high | 3,387,136 | 13,450 | 4.0 | 0.48 (0.47-0.49) |  | 0.35 (0.34-0.36) |  | 0.71 (0.70-0.73) |
|  |  | High | 1,115,254 | 6,692 | 6.0 | 0.72 (0.70-0.74) |  | 0.51 (0.50-0.53) |  | 1.11 (1.07-1.14) |
| **LTSA due to all causes except CMD and MSD** | | |  |  |  |  |  |  |  |  |
|  | Women |  |  |  |  |  |  |  |  |  |
|  |  | Low | 1,429,163 | 30,237 | 21.2 | 1.00 |  | 1.00 |  | 1.00 |
|  |  | Medium-low | 2,515,961 | 64,145 | 25.5 | 1.18 (1.16-1.19) |  | 1.09 (1.07-1.11) |  | 1.21 (1.19-1.23) |
|  |  | Medium-high | 3,524,193 | 77,757 | 22.1 | 1.02 (1.01-1.04) |  | 0.95 (0.94-0.97) |  | 1.30 (1.28-1.33) |
|  |  | High | 3,561,626 | 117,196 | 32.9 | 1.49 (1.47-1.51) |  | 1.37 (1.35-1.39) |  | 1.86 (1.82-1.89) |
|  | Men |  |  |  |  |  |  |  |  |  |
|  |  | Low | 5,592,694 | 116,208 | 20.8 | 1.00 |  | 1.00 |  | 1.00 |
|  |  | Medium-low | 3,786,582 | 67,483 | 17.8 | 0.85 (0.85-0.86) |  | 0.71 (0.70-0.72) |  | 0.91 (0.90-0.92) |
|  |  | Medium-high | 3,387,136 | 39,270 | 11.6 | 0.56 (0.56-0.57) |  | 0.46 (0.46-0.47) |  | 0.78 (0.77-0.79) |
|  |  | High | 1,115,254 | 18,503 | 16.6 | 0.80 (0.78-0.81) |  | 0.64 (0.63-0.65) |  | 1.14 (1.12-1.17) |
|  |  |  |  |  |  |  |  |  |  |  |

Model 1: Unadjusted. Model 2: Adjusted for age. Model 3: Further adjusted for birth country, education, type of living area, family situation and physical demands at work. Abbreviations: LTSA, long-term-sickness absence; CMD, common mental disorder; MSD, musculoskeletal disorder; PY, person-years; HR, hazard ratio; CI, confidence interval.

**e-Figure 1: Emotional demands at work and risk of a new spell of long-term sickness absence exceeding 14 days during 7 years of follow-up**


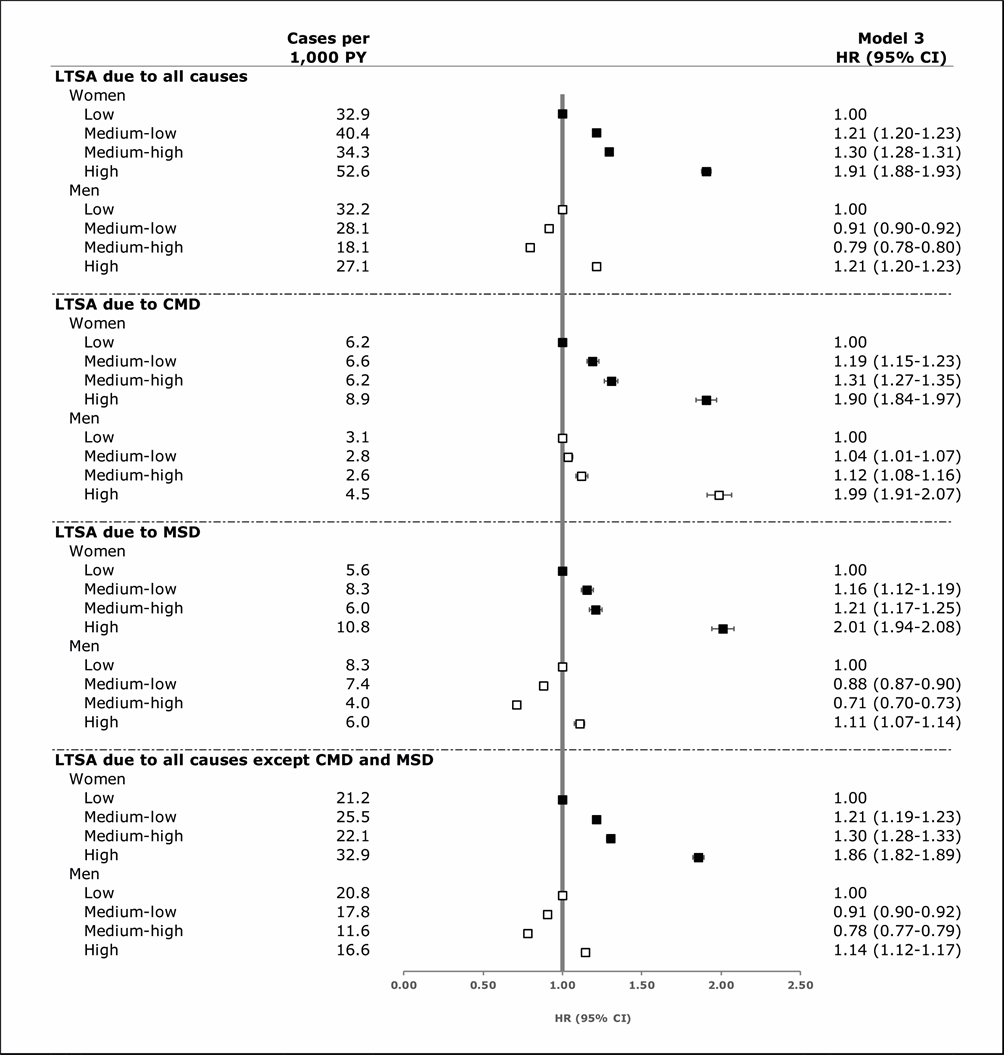


Model 3: Adjusted for age, birth country, education, type of living area, family situation and physical demands at work. Abbreviations: LTSA, long-term-sickness absence; CMD, common mental disorder; MSD, musculoskeletal disorder; PY, person-years; HR, hazard ratio; CI, confidence interval.

**e-Table 2: Emotional demands at work and risk of a new spell of long-term sickness absence exceeding 30 days during 7 years of follow-up without (model 2a) and with (model 3) adjustment for physical demands at work**

|  |  |  | **PY** | **Cases** | **Cases per  1,000 PY** | **Model 2a**  **HR (95% CI)** |  | **Model 3**  **HR (95% CI)** |
| --- | --- | --- | --- | --- | --- | --- | --- | --- |
| **LTSA due to all causes** | | |  |  |  |  |  |  |
|  | Women | |  |  |  |  |  |  |
|  |  | Low | 1,503,561 | 30,063 | 20.0 | 1.00 |  | 1.00 |
|  |  | Medium-low | 2,706,005 | 61,572 | 22.8 | 1.14 (1.12-1.16) |  | 1.23 (1.21-1.25) |
|  |  | Medium-high | 3,771,867 | 69,452 | 18.4 | 1.08 (1.06-1.10) |  | 1.32 (1.30-1.34) |
|  |  | High | 3,930,120 | 108,193 | 27.5 | 1.62 (1.59-1.65) |  | 1.92 (1.88-1.96) |
|  | Men | |  |  |  |  |  |  |
|  |  | Low | 5,896,703 | 114,974 | 19.5 | 1.00 |  | 1.00 |
|  |  | Medium-low | 3,976,768 | 66,309 | 16.7 | 0.80 (0.79-0.81) |  | 0.91 (0.90-0.92) |
|  |  | Medium-high | 3,500,485 | 37,473 | 10.7 | 0.63 (0.62-0.64) |  | 0.82 (0.81-0.83) |
|  |  | High | 1,169,682 | 18,428 | 15.8 | 0.93 (0.91-0.95) |  | 1.23 (1.21-1.25) |
| **LTSA due to CMD** | | |  |  |  |  |  |  |
|  | Women | |  |  |  |  |  |  |
|  |  | Low | 1,503,561 | 6,529 | 4.3 | 1.00 |  | 1.00 |
|  |  | Medium-low | 2,706,005 | 11,942 | 4.4 | 1.20 (1.16-1.25) |  | 1.20 (1.16-1.25) |
|  |  | Medium-high | 3,771,867 | 15,139 | 4.0 | 1.23 (1.19-1.28) |  | 1.31 (1.26-1.37) |
|  |  | High | 3,930,120 | 21,219 | 5.4 | 1.75 (1.68-1.82) |  | 1.82 (1.75-1.90) |
|  | Men | |  |  |  |  |  |  |
|  |  | Low | 5,896,703 | 12,950 | 2.2 | 1.00 |  | 1.00 |
|  |  | Medium-low | 3,976,768 | 7,900 | 2.0 | 1.01 (0.98-1.04) |  | 1.04 (1.01-1.08) |
|  |  | Medium-high | 3,500,485 | 6,201 | 1.8 | 1.08 (1.04-1.12) |  | 1.17 (1.13-1.22) |
|  |  | High | 1,169,682 | 3,632 | 3.1 | 1.85 (1.78-1.93) |  | 2.01 (1.92-2.11) |
| **LTSA due to MSD** | | |  |  |  |  |  |  |
|  | Women | |  |  |  |  |  |  |
|  |  | Low | 1,503,561 | 5,187 | 3.4 | 1.00 |  | 1.00 |
|  |  | Medium-low | 2,706,005 | 13,314 | 4.9 | 1.00 (0.96-1.04) |  | 1.14 (1.09-1.19) |
|  |  | Medium-high | 3,771,867 | 13,391 | 3.6 | 0.87 (0.84-0.91) |  | 1.21 (1.15-1.26) |
|  |  | High | 3,930,120 | 24,335 | 6.2 | 1.43 (1.38-1.50) |  | 1.92 (1.83-2.01) |
|  | Men | |  |  |  |  |  |  |
|  |  | Low | 5,896,703 | 30,736 | 5.2 | 1.00 |  | 1.00 |
|  |  | Medium-low | 3,976,768 | 18,547 | 4.7 | 0.76 (0.75-0.78) |  | 0.88 (0.86-0.90) |
|  |  | Medium-high | 3,500,485 | 9,177 | 2.6 | 0.54 (0.53-0.56) |  | 0.77 (0.74-0.79) |
|  |  | High | 1,169,682 | 4,472 | 3.8 | 0.79 (0.76-0.82) |  | 1.13 (1.08-1.17) |
| **LTSA due to all causes except CMD and MSD** | | | |  |  |  |  |  |
|  | Women | |  |  |  |  |  |  |
|  |  | Low | 1,503,561 | 18,347 | 12.2 | 1.00 |  | 1.00 |
|  |  | Medium-low | 2,706,005 | 36,316 | 13.4 | 1.15 (1.13-1.18) |  | 1.25 (1.22-1.28) |
|  |  | Medium-high | 3,771,867 | 40,922 | 10.8 | 1.09 (1.06-1.11) |  | 1.34 (1.31-1.38) |
|  |  | High | 3,930,120 | 62,639 | 15.9 | 1.62 (1.58-1.65) |  | 1.93 (1.88-1.98) |
|  | Men | |  |  |  |  |  |  |
|  |  | Low | 5,896,703 | 71,288 | 12.1 | 1.00 |  | 1.00 |
|  |  | Medium-low | 3,976,768 | 39,862 | 10.0 | 0.79 (0.78-0.80) |  | 0.89 (0.88-0.91) |
|  |  | Medium-high | 3,500,485 | 22,095 | 6.3 | 0.60 (0.59-0.61) |  | 0.79 (0.77-0.80) |
|  |  | High | 1,169,682 | 10,324 | 8.8 | 0.85 (0.83-0.87) |  | 1.13 (1.11-1.16) |
|  |  |  |  |  |  |  |  |  |

Model 2a: Adjusted for age, birth country, education, type of living area, family situation. Model 3: further adjusted for physical demands at work.
Abbreviations: LTSA, long-term-sickness absence; CMD, common mental disorder; MSD, musculoskeletal disorder; PY, person-years; HR, hazard ratio; CI, confidence interval.
